# Frequency Distribution of COMT Polymorphisms in Greek Patients with Schizophrenia and Controls: A Study of SNPs rs737865, rs4680, and rs165599

**DOI:** 10.5402/2012/651613

**Published:** 2012-11-01

**Authors:** Kotrotsou Maria, Touloumis Charalampos, Dido Vassilakopoulou, Syriou Stavroula, Kalampoki Vasiliki, Drakoulis Nikolaos

**Affiliations:** ^1^Department of Pharmaceutical Technology, School of Pharmacy, National and Kapodistrian University of Athens, Panepistimiopolis, 15771 Athens, Greece; ^2^Department 10th of Dafni-Attica's Psychiatric Hospital, Athinon Avenue 374, Haidari, 12462 Athens, Greece; ^3^Departments of Biochemistry and Molecular Biology, School of Biology, National and Kapodistrian University of Athens, Panepistimiopolis, 15701 Athens, Greece; ^4^Research Diagnostics, Spin-off Company of the University of Athens, Panepistimiopolis, 15771 Athens, Greece

## Abstract

Schizophrenia, a severe psychiatric condition, is characterized by disturbances of cognition, emotion, and social functioning. The disease affects almost 1% of world population. Recent studies evaluating the role of catechol-O-methyltransferase enzyme (COMT) polymorphisms in the pathogenesis of schizophrenia have resulted in ambiguous findings. The current study examined the association of schizophrenia with three COMT polymorphisms, namely, rs737865, rs4680, and rs165599 in a Greek population. There was no significant association between schizophrenia and any of the three SNPs examined. However, haplotype analysis showed that cases have higher frequency of the T-A-A haplotype, and participants with that haplotype were at increased risk for developing schizophrenia (OR = 1.52; CL : 1.12–2.08; *P* = 0.008). Furthermore, patients with schizophrenia displayed an excess of TC/AA/AA and the TT/AA/GA genotypes. Similarly a protective effect of TT/GG/GG and TT/GA/GG was suggested by our results.

## 1. Background

Schizophrenia is a severe, particularly devastating, psychiatric disorder affecting approximately 1% of the general population [1]. The disease is accompanied by significant social dysfunction. The onset of the symptoms usually occurs in young adulthood. Even though schizophrenia is highly heritable, the research for chromosomal loci and candidate genes has not provided any consistent results. Combinations of genetic, epigenetic, and environmental factors participate in the development of the disease. Since these factors have not been identified, the diagnosis of schizophrenia is based on phenotypic symptoms only [2]. Therefore, the identification of susceptibility genes is likely to provide valuable insights into the etiology and pathogenesis of the disease, consequently leading to the development of more effective treatments.

Catechol-O-methyltransferase (COMT) is an enzyme, which catalyses the O-methylation of catecholamine neurotransmitters such as dopamine, adrenaline, and noradrenaline [3].

Disturbances in dopaminergic transmission have long been implicated in schizophrenia [4]. A “reformulated” hypothesis of the dopamine's role in the disease states that hyperdopaminergic functioning in subcortical structures is associated with positive symptoms such as hallucinations and delusions, whereas hypodopaminergic functioning in prefrontal cortical regions is associated with negative and cognitive symptoms [5]. Dopamine is inactivated either by reuptake into the neurons that release dopamine into the synapse or through metabolism by monoamine oxidase or COMT. In most areas of the brain, reuptake inactivation predominates, so that COMT does not markedly influence dopamine levels. By contrast, in the prefrontal cortex, that mediates the cognitive functions, which are impaired in schizophrenia, dopamine levels are sensitive to COMT levels [6]. 

Velocardiofacial syndrome (VCFS) is associated with a microdeletion on chromosome 22q11. Patients with VCFS display an extremely high incidence of schizophrenia about 25% to 30%, and 22q11 deletion occurs in 2% of diagnosed schizophrenics. COMT gene maps to the VCFS region of chromosome 22 [7, 8]. 

Due to its involvement in the catabolic clearance of dopamine and its location in the 22q11 microdeletion area, COMT is a plausible candidate gene for schizophrenia.

The COMT gene is associated with allelic variation [9]. The best-studied variant is a functional single-nucleotide polymorphism, which results in a valine to methionine mutation at position 158 (commonly referred to as Val158Met or rs4680). The val variant has higher enzymatic COMT activity and thermostability than its methionine counterpart, leading to more efficient degradation of dopamine [10]. Therefore, the Val variant is related to poor performance on certain tests of working memory and to insufficient brain activation [11, 12].

 There is weak and inconsistent evidence that the Val variant may be associated with increased risk of schizophrenia [13]. Most case control studies and meta-analysis [14, 15] do not support association, whereas studies using a family design actually do [16].

Other variants, which have been studied for association with schizophrenia, are rs737865 and rs165599.

The present study examines evidence for association of the three SNPs (rs737865, rs4680, and rs165599) and their haplotypes with schizophrenia in a Greek population.

## 2. Subjects and Methods 

### 2.1. Study Subjects

 The study sample consisted of 108 cases with verified schizophrenia and 97 healthy individuals. Blood samples of schizophrenia patients were collected from hospitalized patients in the 10th clinical department of the Attica's Psychiatric hospital “Dafni,” whereas control blood samples were obtained from healthy individuals, who had free anamnesis of schizophrenia and volunteered to participate in this study.  Table 1 shows the distribution of cases and controls by sociodemographic characteristics. Gender distribution and median age were similar in the two groups of participants. The inclusion criteria specified that all patients fulfilled the diagnostic criteria of schizophrenia according to the Diagnostic and Statistical Manual of Mental Disorder (DSM-IV). All participants were of Greek ethnic origin and had signed the informed-consent form. The study was approved by Hospital's Ethic Committee. According to the exclusion criteria, subjects who denied providing informed consent or those whose psychosis was judged secondary to substance use or a known neurological disorder, such as epilepsy, based on the consensus diagnostic procedure, were not eligible to participate. Furthermore, subjects with severe mental retardation were excluded. 

### 2.2. Genotyping Assay

Collection of blood samples was performed in cases as well as controls. Genomic DNA was extracted using Wizard Gemonic DNA Purification kit (Promega). The three SNPs (rs737865, rs4680, and rs165599) were genotyped using PCR real-time analysis on the LightCycler 480 (Roche). LightMix kit (TIB-MOLBIOL), which contained specific primers and probes for 96 PCR-RT chain reactions, and Lightcycler Genotyping 480 DNA Master of Roche Diagnostics, which contains Tag DNA polymerase, reaction buffer, mixture of dNTP (with dATP, dCTP, dGTP, and dUTP) and 15 mM MgCl_2_ were used.

Briefly 20 *μ*L of reaction master mix for COMT gene was performed with H_2_O 10.4 *μ*L, Reagent Mix 1.0 *μ*L, Lightcycler Genotyping 480 DNA Master 2.0 *μ*L, MgCl_2 _1.6 *μ*L, DNA 5.0 *μ*L (~50 ng).

Thermal cycling conditions for PCR were as follows: denaturation at 95°C for 10 minutes, 1 cycle, followed by 45 cycles of 95°C for 10 sec, 60°C for 10 sec and 72°C for 15 sec, followed by melting at 95°C for 30 sec, 40°C for 2 min and 75°C for 1 sec, 1 cycle and cooling at 40°C for 30 sec, 1 cycle.

### 2.3. Statistical Analysis

Deviations from Hardy-Weinberg equilibrium (HWE) were assessed using the chi-square test at each SNP locus separately in cases and controls. To assess the differences in demographic characteristics, we used chi-square for categorical data and *t* test for continuous data. Statistical inference for single, univariate SNP associations in the COMT gene was derived from Fisher's exact test. The odds ratios for dominant, recessive, and additive genetic models for each individual SNP were calculated. The relevant *P* values derived from Wald test for dominant and recessive models and Armitage test for trend for additive model. Subsequently, all possible combinations of the three SNPs were constructed, and thus, differences in haplotype frequencies between schizophrenia patients and control individuals were calculated using Chi-square test. A logistic regression analysis was performed to estimate the risk for schizophrenia of each one of the eight haplotypes. SNP rs737865 carries the alleles T and C and the possible genotypes of that polymorphism are T/T, T/C, or C/C. SNP rs4680 carries the alleles A and G, and its genotypes are A/A, G/A, or G/G. A and G are the two alleles of SNP rs165599, and the possible genotypes are A/A, G/A, or G/G (Table 2). To further explore the combination of genotype is from which arise the T/A/A and T/G/G haplotypes, multivariate logistic regression analysis was performed estimating the risk of schizophrenia in each combination. The SAS statistical package (Version 9.1, SAS Institute Inc, Cary, NC) was used to analyze the data. Significance level was set at *P* = 0.05.

## 3. Results and Discussion

### 3.1. Results

Polymorphism rs4680 was in accordance with Hardy-Weinberg equilibrium (HWE), whereas borderline evidence for deviation from HWE was identified in polymorphism rs165599 among controls (*X* ^2^ = 5.11; *P* = 0.03) and in rs737865 among cases (*X* ^2^ = 4.15; *P* = 0.04). Table 2 shows polymorphism frequencies in patients and controls. None of the three SNPs were found to be independently associated with schizophrenia. All the three genetic models, recessive, dominant, and additive, in the three individual SNPs failed to investigate any evidence for a possible genetic contribution of the SNPs to schizophrenia susceptibility (Table 3).

Further, a haplotype analysis was performed by constructing all the three-site haplotypes that consisted of the alleles of SNPs rs4680, rs165599, and rs737865 (Table 4). The most frequent haplotypes were T/A/A and T/G/G. Significant differences in the frequencies were found between patients and controls regarding T/A/A and T/G/G. Specifically, cases had higher frequency of T/A/A (*P* = 0.010) and lower frequency of T/G/G (*P* = 0.088) as compared to the controls. The remaining six haplotypes had very similar representation in the two groups of participants. When a logistic regression analysis was performed, with schizophrenia as outcome, wild type as the baseline category, and the eight haplotypes as explanatory variables, a significant adverse effect of T/A/A was revealed. Therefore, compared to the baseline expression of T/G/G, participants with T/A/A were found to be associated with 52% higher risk of schizophrenia (OR = 1.52; 95% CI: 1.12–2.08; *P* = 0.008), whereas there was no evidence for association with the psychiatric illness regarding all the other haplotypes. To evaluate further the supporting evidence for the increased risk of T/A/A and for protective effect of T/G/G, a more detailed analysis was performed examining all the possible combinations of genotypes from which the T/A/A and T/G/G haplotypes were derived (Tables 5 and 6). Such analysis showed that after taking the most frequent expression, T/T-A/A-A/A, as baseline, participants with T/C-A/A-A/A were at increased risk for developing the disease (OR = 2.13; 95% CI: 1.02–4.47; *P* = 0.045). Similarly, participants with T/T-A/A-G/A were more likely to develop the disease (OR = 3.20; 95% CI: 1.02–10.05; *P* = 0.046) as compared to those with T/T-A/A-A/A. Regarding T/G/G, we found a protective effect of T/T-G/G-G/G (OR = 0.22; 95% CI: 0.09–0.56; *P* = 0.001) and T/T-G/A-G/G (OR = 0.33; 95% CI: 0.12–0.87; *P* = 0.025) both compared to the baseline, most frequent, category T/T-G/A-G/A. In this multivariate model, two combinations, namely, T/T-G/G-G/A and T/C-G/A-G/G, were excluded given their extremely low frequencies.

## 4. Discussion

Previously, Shifman et al. [17] reported a large study of COMT in schizophrenia, including over 700 patients and 4000 controls. The researchers used Israeli Ashkenazi Jewish population, a well-characterized homogeneous population, in order to reduce genetic variance, and they tested the same SNPs examined in our study, firstly each SNP independently and further the combination of the two SNPs haplotype as well as the three SNPs haplotype. The study showed that in that specific population the association of the Val/Met polymorphism (rs4680) with schizophrenia was modest (*P* = 0.024), whereas the two other polymorphisms rs737865 and rs165599 (located in intron 1 and 3′ flanking region, resp.) were highly significantly associated, as was the haplotype of all three markers G-G-G of rs737865, rs4680, and rs165599 (*P* = 9.6 × 10^−8^). Two independent studies, namely the study of Chen et al. [18] and the study of Handoko et el. [19] have reported a significant association of COMT with schizophrenia. But both of them reported different alleles of the three SNPs consisting the risk haplotype, than the alleles described by Shifman et al. [17]. Specifically, Chen et al. [18] studied the association of COMT with schizophrenia in Irish families with high density of schizophrenia. They genotyped the same SNPs described in Shifman's and in our study. They concluded that Val allele might carry a small increase in disease susceptibility, but they did not confirm any association of rs737865 and rs165599. In our study none of the three SNPs were found to be independently associated with schizophrenia. However, Chen et al.'s hapolype analyses showed that A-G-A haplotype for SNPs rs737865, rs4680, and rs165599 was preferentially transmitted to the affected subjects. This finding was different from the reported G-G-G haplotype found in Ashkenazi Jews and different from the risk haplotype of our study. Handoko et al. [19] studied the above SNPs in a sample of Australian Caucasian families, containing 107 patients with schizophrenia. The haplotype analyses showed that A-A-A combination for SNPs rs737865, rs4680, and rs165599 was the risk haplotype for the studied population. That haplotype was in accordance with the risk haplotype found in our study in Greek population.

Analytically, in the present study the allele frequency of COMT Val/Met polymorphism was typical to the European population, since in Europe the two alleles are very similar in frequency [20]. There was no significant association between schizophrenia and any of the three alleles.

Furthermore, a three-marker haplotype analysis was performed, consisting of combinations of the putative functional SNPs. In this study, cases have higher frequency of the rs737865/rs4680/rs165599 T-A-A haplotype and lower frequency of T-G-G haplotype. G is the ancestral allele of rs4680 and rs165599. The C allele at rs737865 corresponds to the G allele in the study of Shifman et al. [17], and the T allele, which refers to the A allele, is the wild-type allele of rs737865 polymorphism. The T-G-G haplotype, which consists of the three wild types alleles of polymorphisms rs737865, rs4680, and rs165599, is present at reasonably high frequencies in controls.

Furthermore, this study shows that compared to the baseline expression T-G-G, participants with T-A-A haplotype were found to be associated with 52% higher risk of schizophrenia (Table 4).

Conclusively, among studies of European subjects, which examined the same SNPs described in the present study, different populations have been reported to exhibit an association between schizophrenia and the different triple SNP haplotypes. As already mentioned, examples of this include the G-G-G haplotype in Ashkenazi Jews [17], the A-G-A haplotype in Irish families [18], the A-G-G haplotype which was significantly under transmitted in Australian family-affected samples [19], and the protective haplotype A-A-A in a case-control study of Caucasian subjects [21]. The high-risk haplotype in the Ashkenazi sample was actually under transmitted to affected individuals in our sample. The study of Shifman et al. [17] is the first and largest population-based association study to date to find a significant association between COMT haplotype, constructed by the three SNPs, and schizophrenia. The Ashkenazi Jewish population may not be directly comparable with other European populations since this haplotype may possibly contribute risk to schizophrenia in a different pathway across multiple ethnic populations [22]. Furthermore, the phenotypic complexity of the disorder makes it difficult to achieve a great clinical homogeneity in the sample of patients. The diagnosis of the disease is based upon clinical phenomena, which may not correctly reflect the underlying biology that predisposes to illness and the absence of biological markers limits the homogeneity of diagnosis. One approach that has been suggested is to subdivide schizophrenia into components of symptom complexes and to develop targets of narrower clinical features [23].

Furthermore, all possible genotype combinations of the risk haplotype T-A-A were examined. Patients with schizophrenia displayed an excess of the TC-AA-AA genotype homozygous for the two mutant alleles and the TT-AA-GA genotype, homozygous for the mutant rs4680 allele and heterozygous for rs165599. Similarly, a protective effect of genotypes TT-GG-GG and TT-GA-GG was documented.

In addition, Bray et al. [24], showed that Shifman's risk haplotype [17] G-G-G (C-G-G) is associated with low COMT mRNA expression in prefrontal cortex. It is possible that the opposite haplotype A-A-A (T-A-A), which is the risk haplotype in this study confers high COMT mRNA expression. This speculation is compatible with the reformulated theory of hypofrontality in schizophrenia [5, 11].

## 5. Conclusion 

The current study shows an association of COMT gene and schizophrenia in a Greek population. The haplotype analysis indicates that the T-A-A haplotype had high association with schizophrenia.

## Figures and Tables

**Table 1:** Demographic characteristics in 108 schizophrenia patients and 97 controls.

Variables	Patients (*n* = 108)	Controls (*n* = 97)	*P* value^†^
Gender			
Male	69 (63.9)	71 (73.2)	0.153
Female	39 (36.1)	26 (26.8)	
Median Age, yrs	43.32 ± 12.24	42 ± 11.68	0.430

^†^ *P* values derived from *t*-test for age; chi-square test was used for gender.

**Table 2:** Polymorphisms frequencies in 108 schizophrenia patients and 97 controls.

SNPs	Patients (*n* = 108)	Controls (*n* = 97)	*P* value^†^
rs737865			
T/C	57 (52.8)	51 (52.6)	
T/T	44 (40.7)	37 (38.1)	0.787
C/C	7 (6.5)	9 (9.3)	
rs4680			
G/A	56 (51.9)	55 (56.7)	
A/A	24 (22.2)	15 (15.5)	0.511
G/G	28 (25.9)	27 (27.8)	
rs165599			
G/A	61 (56.5)	59 (60.8)	
A/A	34 (31.5)	24 (24.7)	0.558
G/G	13 (12.0)	14 (14.5)	

Data are presented as *N* (%).

^†^Fisher's exact test was used for genotypes.

**Table 3:** Single SNP Analysis: logistic regression-derived, crude odds ratios (OR), 95% confidence intervals (95% CI), and *P* values for the risk of schizophrenia in each SNP (rs737865, rs4680, and rs165599) according to the recessive, dominant, additive genetic models, and analysis per allele in the total of 205 participants.

Polymorphisms	Crude odds ratio analysis		
	OR	95% CI	*P* value
rs**737865**			
Recessive model			
CC versus TT + TC	0.68	0.24–1.90	0.459*
Dominant model			
CC + TC versus TT	0.90	0.51–1.57	0.705*
Additive model			
C allele versus TT	0.87	0.55–1.36	0.528**
Analysis per allele			
C allele versus T allele	0.89	0.59–1.34	0.565*

rs**4680**			
Recessive model			
AA versus GG + GA	1.56	0.77–3.19	0.221*
Dominant model			
AA + GA versus GG	1.10	0.59–2.05	0.758*
Additive model			
A allele versus GG	1.21	0.81–1.83	0.358**
Analysis per allele			
A allele versus G allele	1.19	0.81–1.76	0.380*

rs**165599**			
Recessive model			
AA versus GG + GA	1.40	0.76–2.58	0.286*
Dominant model			
AA + GA versus GG	1.23	0.55–2.77	0.612*
Additive model			
A allele versus GG	1.26	0.81–1.96	0.298**
Analysis per allele			
A allele versus G allele	1.21	0.81–1.79	0.350*

*P values derived from Wald test.

**P values derived from Armitage test for trend.

**Table 4:** Haplotype analysis: frequencies and logistic regression-derived odds ratios (OR) for the risk of schizophrenia in the study participants.

Haplotypes rs737865/4680/165599	Patients *N* (%)	Controls *N* (%)	OR	95% CI	*P* value^†^
T/G/G	133 (15.5)	144 (18.6)	Baseline	—	—
T/A/A	225 (26.0)	160 (20.6)	1.52	1.12–2.08	**0.008**
T/G/A	139 (16.1)	118 (15.2)	1.28	0.91–1.79	0.161
C/G/G	103 (11.9)	90 (11.6)	1.24	0.86–1.79	0.254
C/G/A	77 (8.9)	84 (10.8)	0.99	0.67–1.46	0.970
T/A/G	83 (9.6)	78 (10.1)	1.15	0.78–1.70	0.475
C/A/A	71 (8.2)	66 (8.5)	1.17	0.77–1.76	0.466
C/A/G	33 (3.8)	36 (4.6)	0.99	0.59–1.68	0.978

^†^ *P* values derived from Wald test.

**Table 5:** Frequencies of all possible combinations of genotypes from which arises the T/A/A haplotype and logistic regression-derived odds ratios (OR) for the risk of schizophrenia.

rs737865	rs4680	rs165599	Patients *N*(%)	Controls *N*(%)	Total *N* (%)	OR	95% CI	*P* value^†^
T/T	A/A	A/A	80 (35.6)	64 (40.0)	144 (37.4)	Baseline	—	—
T/T	G/A	G/A	32 (14.2)	26 (16.2)	58 (15.1)	0.99	0.53–11.82	0.961
T/C	G/A	G/A	21 (9.4)	24 (15.0)	45 (11.7)	0.70	0.36–1.37	0.298
T/T	G/A	A/A	32 (14.2)	16 (10.0)	48 (12.5)	1.60	0.81–3.17	0.178
**T/C**	**A/A**	**A/A**	**32 (14.2)**	**12 (7.5)**	**44 (11.4)**	**2.13**	**1.02–4.47**	**0.045**
T/C	G/A	A/A	10 (4.4)	12 (7.5)	22 (5.7)	0.67	0.27–1.64	0.378
**T/T**	**A/A**	**G/A**	**16 (7.1)**	**4 (2.5)**	**20 (5.2)**	**3.20**	**1.02–10.05**	**0.046**
T/C	A/A	G/A	2 (0.9)	2 (1.3)	4 (1.0)	0.80	0.11–5.84	0.826

^†^ *P* values derived from Wald test.

**Table 6:** Frequencies of all possible combinations of T/G/G and logistic regression-derived odds ratios (OR) for the risk of schizophrenia.

rs737865	rs4680	rs165599	Patients *N* (%)	Controls *N* (%)	Total *N* (%)	OR	95% CI	*P* value^†^
T/T	G/A	G/A	40 (30.1)	26 (18.0)	66 (23.7)	Baseline	—	—
T/C	G/G	G/G	28 (21.1)	16 (11.1)	44 (15.9)	1.14	0.52–2.50	0.749
T/C	G/A	G/A	21 (15.7)	24 (16.7)	45 (16.3)	0.57	0.26–1.22	0.149
T/C	G/G	G/A	24 (18.1)	20 (13.9)	44 (15.9)	0.78	0.36–1.69	0.528
**T/T**	**G/G**	**G/G**	**8 (6.0)**	**24 (16.7)**	**32 (11.6)**	**0.22**	**0.09–0.56**	**0.001**
**T/T**	**G/A**	**G/G**	**8 (6.0)**	**16 (11.1)**	**24 (8.7)**	**0.33**	**0.12–0.87**	**0.025**
T/T	G/G	G/A	4 (3.0)	16 (11.1)	20 (7.2)	n/a	n/a	n/a
T/C	G/A	G/G	0 (0.0)	2 (1.4)	2 (0.7)	n/a	n/a	n/a

^†^ *P* values derived from Wald test.

Due to small frequencies, the combinations T/T-G/G-G/A and T/C-G/A-G/G were not included in the model.
